# Poly[[[μ_3_-(*E*)-*N*-(pyridin-4-yl­methyl­idene)hy­droxy­laminato-κ^3^ *O*:*N*:*N*′][μ_2_-(*E*)-*N*-(pyridin-4-yl­methyl­idene)hydroxyl­amine-κ^2^ *N*:*N*′][(*E*)-*N*-(pyridin-4-yl­methyl­idene-κ*N*)hydroxyl­amine]­disilver(I)] nitrate]

**DOI:** 10.1107/S1600536812046090

**Published:** 2012-11-28

**Authors:** Shan Gao, Seik Weng Ng

**Affiliations:** a Key Laboratory of Functional Inorganic Material Chemistry, Ministry of Education, Heilongjiang University, Harbin 150080, People’s Republic of China; b Department of Chemistry, University of Malaya, 50603 Kuala Lumpur, Malaysia; c Chemistry Department, Faculty of Science, King Abdulaziz University, PO Box 80203 Jeddah, Saudi Arabia

## Abstract

The title coordination polymer, {[Ag_2_(C_6_H_5_N_2_O)(C_6_H_6_N_2_O)_2_]NO_3_}_*n*_, features a deprotonated *N*-(pyridin-4-yl­methyl­idene)hy­droxy­laminate anion and two neutral *N*-(pyridin-4-yl­methyl­idene)hydroxyl­amine mol­ecules in the asymmetric unit. The anion connects three Ag^I^ atoms through its O and two N-donor atoms. One neutral ligand functions in a monodentate mode; the other functions in a bridging mode, binding though its two N atoms. The coordination geometry of the two independent metal atoms is *T*-shaped; the manner of bridging gives rise to a layer motif parallel to (100). In the crystal, the nitrate ion is disordered over two positions in a 1:1 ratio, and is sandwiched between adjacent layers. O—H⋯O hydrogen bonding is present between nitrate ions and layers, and also between adjacent layers.

## Related literature

For mononuclear (Nitrato-κ^2^ *O*,*O*′)bis­[(*E*)-*N*-(pyridin-4-yl­meth­yl­idene-κ*N*)hy­droxy­amine]­silver(I), see: Gao & Ng (2012 ▶).
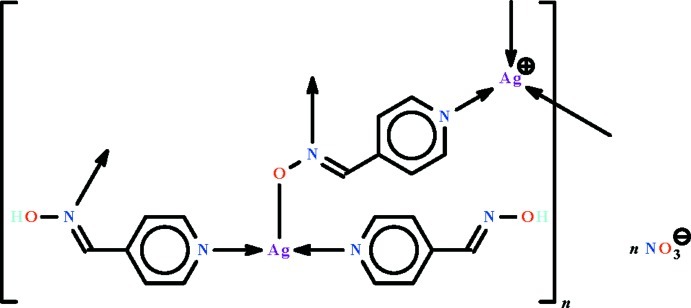


## Experimental

### 

#### Crystal data

[Ag_2_(C_6_H_5_N_2_O)(C_6_H_6_N_2_O)_2_]NO_3_
*M* *_r_* = 643.13
Monoclinic, 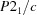

*a* = 13.1628 (18) Å
*b* = 10.9926 (18) Å
*c* = 16.315 (2) Å
β = 110.412 (4)°
*V* = 2212.4 (6) Å^3^
*Z* = 4
Mo *K*α radiation
μ = 1.82 mm^−1^
*T* = 293 K
0.20 × 0.12 × 0.12 mm

#### Data collection

Rigaku R-AXIS RAPID IP diffractometer
Absorption correction: multi-scan (*ABSCOR*; Higashi, 1995 ▶) *T*_min_ = 0.712, *T*_max_ = 0.811
21226 measured reflections
5047 independent reflections
3355 reflections with *I* > 2σ(*I*)
*R*_int_ = 0.047

#### Refinement

*R*[*F* ^2^ > 2σ(*F* ^2^)] = 0.042
*wR*(*F* ^2^) = 0.119
*S* = 1.07
5047 reflections
306 parameters
26 restraints
H-atom parameters constrained
Δρ_max_ = 0.77 e Å^−3^
Δρ_min_ = −0.43 e Å^−3^

### 

Data collection: *RAPID-AUTO* (Rigaku, 1998 ▶); cell refinement: *RAPID-AUTO*; data reduction: *CrystalClear* (Rigaku/MSC, 2002 ▶); program(s) used to solve structure: *SHELXS97* (Sheldrick, 2008 ▶); program(s) used to refine structure: *SHELXL97* (Sheldrick, 2008 ▶); molecular graphics: *X-SEED* (Barbour, 2001 ▶); software used to prepare material for publication: *publCIF* (Westrip, 2010 ▶).

## Supplementary Material

Crystal structure: contains datablock(s) global, I. DOI: 10.1107/S1600536812046090/xu5648sup1.cif

Structure factors: contains datablock(s) I. DOI: 10.1107/S1600536812046090/xu5648Isup2.hkl

Additional supplementary materials:  crystallographic information; 3D view; checkCIF report

## Figures and Tables

**Table 1:** Selected bond lengths (Å)

Ag1—O1	2.612 (3)
Ag1—N3	2.151 (3)
Ag1—N5	2.158 (3)
Ag2—O1^i^	2.546 (2)
Ag2—N1	2.161 (3)
Ag2—N2^ii^	2.183 (3)

Symmetry codes: (i) 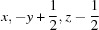; (ii) 
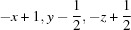
.

**Table 2:** Hydrogen-bond geometry (Å, °)

*D*—H⋯*A*	*D*—H	H⋯*A*	*D*⋯*A*	*D*—H⋯*A*
O2—H2⋯O4	0.84	1.81	2.642 (9)	169
O2—H2⋯O4′	0.84	2.21	2.997 (14)	156
O3—H3⋯O1^iii^	0.84	1.69	2.527 (4)	174

Symmetry code: (iii) 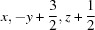.

**Fig. 1.**
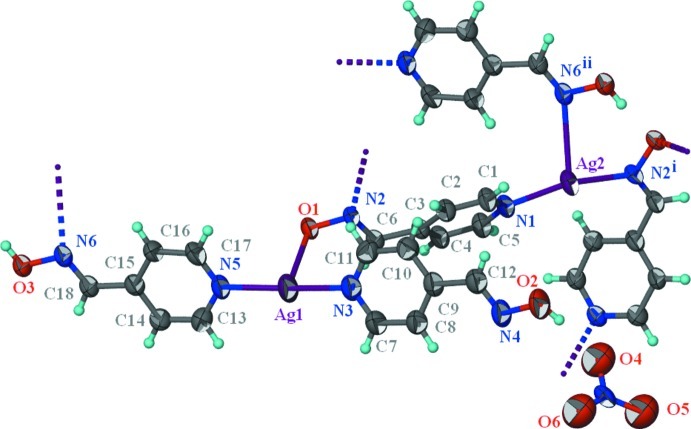
Thermal ellipsoid plot (Barbour, 2001) of polymeric [Ag2(C6H5N2O)(C6H6N2O)2] NO3 at the 50% probability level; hydrogen atoms are drawn as spheres of arbitrary radius. The disorder in the nitrate is not shown.

**Fig. 2.**
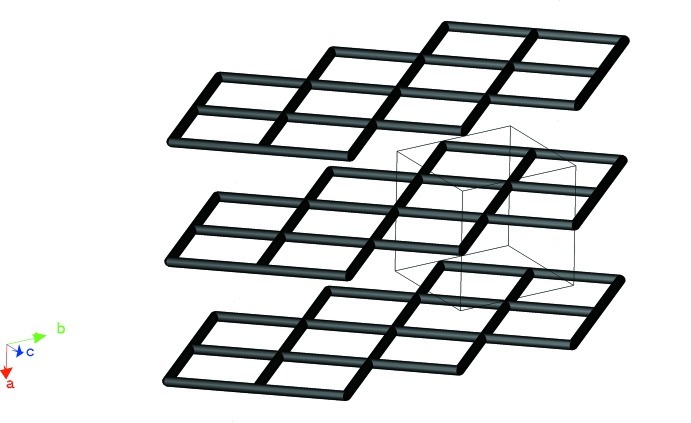
Layer motif.

## supplementary crystallographic information

### Comment

The preceding report presents the crystal structure of bis(isonicotinylaldehyde oxide)(nitrato)silver (Gao & Ng, 2012). When some ammonium hydroxide was added to the reaction, the acid hydrogen of the isonicotinylaldehyde oxide is partially removed so that the product consists of a cation having two AgI atoms, one deprotonated ligand and two neutral ligands. The charge is balanced by a nitrate anion. The dinuclear cations are linked by coordination bonds into a polycation (Scheme I). The coordination polymer features a deprotonated *N*-(pyridin-4-ylmethylidene)hydroxylamine and two neutral *N*-(pyridin-4-ylmethylidene)hydroxylamine molecules. The anion connects three Ag^I^ atoms through its O and two N donor-atoms. One neutral ligand functions in a monodentate mode; the other functions in a bridging mode, binding though its two N atoms. The geometry of the two independent metal atoms is *T*-shaped (Fig. 1); the manner of bridge gives rise to a layer motif. The anion is sandwiched between adjacent layers (Fig. 2).

### Experimental

Isonicotinaldehyde oxime was synthesized from the reaction of isonicotinaldehyde and hydroxylamine. Silver nitrate (1 mmol) dissolved in water (5 ml) was added to picolinaldehyde oxime (1 mmol) dissolved in ethanol (5 ml). Several drops of ammonium hydroxide were added. The solution was filtered and set aside, away from light, for the growth of colorless crystals.

### Refinement

Carbon- and oxygen-bound H-atoms were placed in calculated positions (C–H 0.93 Å, O–H 0.84 Å) and were included in the refinement in the riding model approximation, with *U*(H) set to 1.2–1.5*U*(C,*O*).

The nitrate ion is disordered over two positions in a 1:1 ratio. Nitrogen–oxygen distances were restrained to 1.24±0.01 A and oxygen···oxygen contacts to 2.15±0.01 Å. For each component, the temperature factors of the O atoms were made identical. The temperature factors of the N atoms of the two components were made identical too. The isotropic temperature factors were tightly restrained.

The (3 9 9), (-7 10 13) and (-3 7 18) reflections were omitted.

### Crystal data

**Table:**

[Ag_2_(C_6_H_5_N_2_O)(C_6_H_6_N_2_O)_2_]NO_3_	*F*(000) = 1264
*M**_r_* = 643.13	*D*_x_ = 1.931 Mg m^−^^3^
Monoclinic, *P*2_1_/*c*	Mo *K*α radiation, λ = 0.71073 Å
Hall symbol: -P 2ybc	Cell parameters from 10662 reflections
*a* = 13.1628 (18) Å	θ = 3.1–27.5°
*b* = 10.9926 (18) Å	µ = 1.82 mm^−^^1^
*c* = 16.315 (2) Å	*T* = 293 K
β = 110.412 (4)°	Prism, colorless
*V* = 2212.4 (6) Å^3^	0.20 × 0.12 × 0.12 mm
*Z* = 4	

### Data collection

**Table:**

Rigaku R-AXIS RAPID IP diffractometer	5047 independent reflections
Radiation source: fine-focus sealed tube	3355 reflections with *I* > 2σ(*I*)
Graphite monochromator	*R*_int_ = 0.047
ω scan	θ_max_ = 27.5°, θ_min_ = 3.1°
Absorption correction: multi-scan (*ABSCOR*; Higashi, 1995)	*h* = −17→17
*T*_min_ = 0.712, *T*_max_ = 0.811	*k* = −14→14
21226 measured reflections	*l* = −19→21

### Refinement

**Table:**

Refinement on *F*^2^	Primary atom site location: structure-invariant direct methods
Least-squares matrix: full	Secondary atom site location: difference Fourier map
*R*[*F*^2^ > 2σ(*F*^2^)] = 0.042	Hydrogen site location: inferred from neighbouring sites
*wR*(*F*^2^) = 0.119	H-atom parameters constrained
*S* = 1.07	*w* = 1/[σ^2^(*F*_o_^2^) + (0.0609*P*)^2^] where *P* = (*F*_o_^2^ + 2*F*_c_^2^)/3
5047 reflections	(Δ/σ)_max_ = 0.001
306 parameters	Δρ_max_ = 0.77 e Å^−^^3^
26 restraints	Δρ_min_ = −0.43 e Å^−^^3^

### Fractional atomic coordinates and isotropic or equivalent isotropic displacement parameters (Å^2^)

**Table:**

	*x*	*y*	*z*	*U*_iso_*/*U*_eq_	Occ. (<1)
Ag1	0.82406 (3)	0.44988 (5)	0.68283 (2)	0.0906 (2)	
Ag2	0.45572 (3)	0.01686 (3)	0.114909 (19)	0.06273 (15)	
O1	0.6234 (2)	0.4403 (3)	0.57575 (15)	0.0523 (6)	
O2	1.0680 (3)	0.1718 (4)	0.3037 (2)	0.1041 (13)	
H2	1.0946	0.1023	0.3041	0.156*	
O3	0.6612 (3)	0.8349 (3)	1.07189 (16)	0.0633 (8)	
H3	0.6532	0.9104	1.0744	0.095*	
O4	1.1249 (5)	−0.0582 (8)	0.3006 (6)	0.1012 (16)	0.50
O5	1.2395 (7)	−0.1874 (9)	0.2869 (8)	0.1012 (16)	0.50
O6	1.2906 (5)	−0.0465 (7)	0.3833 (5)	0.1012 (16)	0.50
O4'	1.1788 (14)	−0.0670 (13)	0.3637 (8)	0.187 (4)	0.50
O5'	1.2614 (15)	−0.1635 (11)	0.2915 (11)	0.187 (4)	0.50
O6'	1.2181 (13)	0.0236 (10)	0.2655 (8)	0.187 (4)	0.50
N1	0.5108 (3)	0.0936 (3)	0.24501 (19)	0.0556 (8)	
N2	0.6020 (2)	0.3906 (3)	0.49497 (17)	0.0460 (7)	
N3	0.8829 (3)	0.3913 (4)	0.5817 (2)	0.0707 (11)	
N4	1.0390 (3)	0.1859 (5)	0.3770 (2)	0.0763 (12)	
N5	0.7900 (3)	0.5396 (4)	0.7881 (2)	0.0676 (10)	
N6	0.6741 (3)	0.8056 (3)	0.99397 (19)	0.0522 (8)	
N7	1.2216 (8)	−0.0974 (7)	0.3251 (6)	0.0797 (19)	0.50
N7'	1.2207 (7)	−0.0713 (10)	0.3065 (6)	0.0797 (19)	0.50
C1	0.5737 (3)	0.1916 (4)	0.2613 (2)	0.0578 (10)	
H1	0.5978	0.2198	0.2175	0.069*	
C2	0.6052 (3)	0.2537 (4)	0.3395 (2)	0.0553 (9)	
H2A	0.6491	0.3223	0.3475	0.066*	
C3	0.5710 (3)	0.2131 (4)	0.4062 (2)	0.0510 (9)	
C4	0.5075 (4)	0.1096 (4)	0.3898 (2)	0.0589 (10)	
H4	0.4840	0.0783	0.4331	0.071*	
C5	0.4786 (4)	0.0526 (4)	0.3098 (3)	0.0627 (11)	
H5	0.4354	−0.0166	0.3001	0.075*	
C6	0.5966 (3)	0.2758 (4)	0.4903 (2)	0.0536 (9)	
H6	0.6089	0.2305	0.5410	0.064*	
C7	0.9441 (4)	0.2916 (5)	0.5886 (3)	0.0783 (14)	
H7	0.9633	0.2467	0.6401	0.094*	
C8	0.9799 (4)	0.2525 (5)	0.5230 (3)	0.0720 (13)	
H8	1.0224	0.1830	0.5305	0.086*	
C9	0.9516 (3)	0.3179 (4)	0.4458 (3)	0.0614 (11)	
C10	0.8877 (3)	0.4200 (5)	0.4382 (3)	0.0651 (11)	
H10	0.8670	0.4666	0.3874	0.078*	
C11	0.8553 (4)	0.4518 (5)	0.5069 (3)	0.0701 (13)	
H11	0.8114	0.5200	0.5005	0.084*	
C12	0.9898 (4)	0.2858 (5)	0.3741 (3)	0.0706 (12)	
H12	0.9781	0.3383	0.3270	0.085*	
C13	0.7989 (4)	0.4791 (5)	0.8613 (3)	0.0721 (13)	
H13	0.8249	0.3997	0.8674	0.087*	
C14	0.7717 (4)	0.5284 (4)	0.9274 (3)	0.0623 (11)	
H14	0.7795	0.4827	0.9772	0.075*	
C15	0.7327 (3)	0.6453 (4)	0.9210 (2)	0.0528 (9)	
C16	0.7235 (3)	0.7088 (4)	0.8454 (2)	0.0642 (11)	
H16	0.6980	0.7885	0.8378	0.077*	
C17	0.7525 (4)	0.6524 (5)	0.7817 (3)	0.0730 (13)	
H17	0.7454	0.6960	0.7311	0.088*	
C18	0.7078 (3)	0.6971 (4)	0.9947 (2)	0.0563 (10)	
H18	0.7171	0.6489	1.0437	0.068*	

### Atomic displacement parameters (Å^2^)

**Table:**

	*U*^11^	*U*^22^	*U*^33^	*U*^12^	*U*^13^	*U*^23^
Ag1	0.0834 (3)	0.1332 (5)	0.0585 (2)	0.0248 (2)	0.0289 (2)	−0.0216 (2)
Ag2	0.0788 (3)	0.0733 (3)	0.03828 (19)	−0.01131 (17)	0.02310 (17)	−0.01213 (14)
O1	0.0644 (15)	0.0577 (17)	0.0356 (13)	−0.0003 (13)	0.0187 (12)	−0.0058 (12)
O2	0.111 (3)	0.147 (4)	0.067 (2)	0.034 (3)	0.046 (2)	−0.015 (2)
O3	0.0896 (19)	0.066 (2)	0.0418 (14)	0.0126 (17)	0.0320 (14)	0.0045 (13)
O4	0.079 (3)	0.101 (4)	0.109 (3)	0.025 (2)	0.014 (3)	−0.034 (3)
O5	0.079 (3)	0.101 (4)	0.109 (3)	0.025 (2)	0.014 (3)	−0.034 (3)
O6	0.079 (3)	0.101 (4)	0.109 (3)	0.025 (2)	0.014 (3)	−0.034 (3)
O4'	0.258 (10)	0.189 (9)	0.143 (6)	0.063 (7)	0.107 (7)	0.017 (6)
O5'	0.258 (10)	0.189 (9)	0.143 (6)	0.063 (7)	0.107 (7)	0.017 (6)
O6'	0.258 (10)	0.189 (9)	0.143 (6)	0.063 (7)	0.107 (7)	0.017 (6)
N1	0.069 (2)	0.062 (2)	0.0371 (16)	−0.0044 (18)	0.0200 (15)	−0.0061 (15)
N2	0.0533 (16)	0.054 (2)	0.0300 (14)	0.0030 (15)	0.0140 (13)	−0.0019 (13)
N3	0.064 (2)	0.093 (3)	0.054 (2)	0.009 (2)	0.0206 (18)	−0.014 (2)
N4	0.072 (2)	0.105 (4)	0.055 (2)	0.009 (2)	0.0257 (19)	−0.019 (2)
N5	0.071 (2)	0.086 (3)	0.047 (2)	0.012 (2)	0.0214 (18)	−0.0107 (19)
N6	0.0588 (18)	0.061 (2)	0.0401 (16)	−0.0009 (16)	0.0212 (15)	−0.0022 (15)
N7	0.098 (3)	0.102 (5)	0.050 (4)	0.016 (3)	0.039 (3)	0.003 (3)
N7'	0.098 (3)	0.102 (5)	0.050 (4)	0.016 (3)	0.039 (3)	0.003 (3)
C1	0.076 (3)	0.061 (3)	0.042 (2)	−0.006 (2)	0.027 (2)	−0.0037 (18)
C2	0.068 (2)	0.052 (2)	0.046 (2)	−0.006 (2)	0.0211 (19)	−0.0028 (18)
C3	0.065 (2)	0.049 (2)	0.0373 (17)	0.0062 (19)	0.0171 (17)	−0.0002 (16)
C4	0.082 (3)	0.058 (3)	0.042 (2)	−0.005 (2)	0.027 (2)	0.0007 (18)
C5	0.085 (3)	0.058 (3)	0.051 (2)	−0.014 (2)	0.030 (2)	−0.009 (2)
C6	0.072 (2)	0.053 (3)	0.0359 (18)	0.007 (2)	0.0188 (18)	0.0028 (17)
C7	0.083 (3)	0.098 (4)	0.053 (2)	0.020 (3)	0.023 (2)	−0.002 (3)
C8	0.068 (3)	0.088 (4)	0.059 (2)	0.015 (2)	0.022 (2)	−0.008 (3)
C9	0.048 (2)	0.081 (3)	0.055 (2)	−0.002 (2)	0.0171 (19)	−0.012 (2)
C10	0.057 (2)	0.083 (3)	0.057 (2)	0.007 (2)	0.022 (2)	−0.003 (2)
C11	0.057 (2)	0.078 (3)	0.072 (3)	0.010 (2)	0.018 (2)	−0.014 (3)
C12	0.068 (3)	0.088 (4)	0.057 (3)	0.008 (3)	0.023 (2)	−0.007 (2)
C13	0.077 (3)	0.068 (3)	0.072 (3)	0.011 (2)	0.027 (3)	−0.011 (2)
C14	0.080 (3)	0.060 (3)	0.052 (2)	0.003 (2)	0.028 (2)	0.004 (2)
C15	0.050 (2)	0.062 (3)	0.046 (2)	−0.0010 (18)	0.0171 (17)	−0.0025 (19)
C16	0.074 (3)	0.073 (3)	0.046 (2)	0.016 (2)	0.021 (2)	0.002 (2)
C17	0.085 (3)	0.092 (4)	0.043 (2)	0.015 (3)	0.023 (2)	0.002 (2)
C18	0.069 (2)	0.061 (3)	0.042 (2)	0.002 (2)	0.0229 (19)	0.0016 (18)

### Geometric parameters (Å, º)

**Table:**

Ag1—O1	2.612 (3)	C1—H1	0.9300
Ag1—N3	2.151 (3)	C2—C3	1.389 (5)
Ag1—N5	2.158 (3)	C2—H2A	0.9300
Ag2—O1^i^	2.546 (2)	C3—C4	1.382 (6)
Ag2—N1	2.161 (3)	C3—C6	1.466 (5)
Ag2—N2^ii^	2.183 (3)	C4—C5	1.377 (5)
O1—N2	1.362 (3)	C4—H4	0.9300
O1—Ag2^iii^	2.546 (2)	C5—H5	0.9300
O2—N4	1.387 (4)	C6—H6	0.9300
O2—H2	0.8400	C7—C8	1.381 (6)
O3—N6	1.378 (4)	C7—H7	0.9300
O3—H3	0.8400	C8—C9	1.383 (6)
O4—N7	1.270 (9)	C8—H8	0.9300
O5—N7	1.234 (6)	C9—C10	1.381 (6)
O6—N7	1.199 (11)	C9—C12	1.469 (6)
O4'—N7'	1.239 (7)	C10—C11	1.377 (6)
O5'—N7'	1.211 (8)	C10—H10	0.9300
O6'—N7'	1.234 (8)	C11—H11	0.9300
N1—C1	1.327 (5)	C12—H12	0.9300
N1—C5	1.346 (5)	C13—C14	1.362 (6)
N2—C6	1.264 (5)	C13—H13	0.9300
N2—Ag2^iv^	2.183 (3)	C14—C15	1.374 (6)
N3—C11	1.324 (6)	C14—H14	0.9300
N3—C7	1.342 (6)	C15—C16	1.386 (5)
N4—C12	1.267 (6)	C15—C18	1.467 (5)
N5—C17	1.325 (6)	C16—C17	1.374 (6)
N5—C13	1.337 (6)	C16—H16	0.9300
N6—C18	1.271 (5)	C17—H17	0.9300
C1—C2	1.378 (5)	C18—H18	0.9300
			
N3—Ag1—N5	167.71 (16)	N1—C5—C4	122.3 (4)
N3—Ag1—O1	91.45 (11)	N1—C5—H5	118.9
N5—Ag1—O1	96.21 (12)	C4—C5—H5	118.9
N1—Ag2—N2^ii^	162.98 (12)	N2—C6—C3	121.0 (3)
N1—Ag2—O1^i^	98.67 (10)	N2—C6—H6	119.5
N2^ii^—Ag2—O1^i^	89.75 (9)	C3—C6—H6	119.5
N2—O1—Ag2^iii^	114.36 (19)	N3—C7—C8	123.1 (5)
N2—O1—Ag1	118.84 (18)	N3—C7—H7	118.5
Ag2^iii^—O1—Ag1	125.99 (9)	C8—C7—H7	118.5
N4—O2—H2	109.5	C7—C8—C9	119.2 (5)
N6—O3—H3	109.5	C7—C8—H8	120.4
C1—N1—C5	117.4 (3)	C9—C8—H8	120.4
C1—N1—Ag2	119.0 (2)	C10—C9—C8	117.8 (4)
C5—N1—Ag2	123.4 (3)	C10—C9—C12	119.4 (4)
C6—N2—O1	116.5 (3)	C8—C9—C12	122.8 (4)
C6—N2—Ag2^iv^	126.0 (3)	C11—C10—C9	119.0 (4)
O1—N2—Ag2^iv^	115.8 (2)	C11—C10—H10	120.5
C11—N3—C7	116.9 (4)	C9—C10—H10	120.5
C11—N3—Ag1	120.0 (3)	N3—C11—C10	124.0 (4)
C7—N3—Ag1	123.1 (3)	N3—C11—H11	118.0
C12—N4—O2	110.8 (4)	C10—C11—H11	118.0
C17—N5—C13	116.7 (4)	N4—C12—C9	119.4 (5)
C17—N5—Ag1	122.7 (3)	N4—C12—H12	120.3
C13—N5—Ag1	120.4 (3)	C9—C12—H12	120.3
C18—N6—O3	111.4 (3)	N5—C13—C14	123.0 (5)
O6—N7—O5	123.3 (8)	N5—C13—H13	118.5
O6—N7—O4	119.3 (7)	C14—C13—H13	118.5
O5—N7—O4	117.4 (9)	C13—C14—C15	120.4 (4)
O5'—N7'—O6'	121.9 (8)	C13—C14—H14	119.8
O5'—N7'—O4'	122.0 (9)	C15—C14—H14	119.8
O6'—N7'—O4'	116.2 (8)	C14—C15—C16	117.0 (4)
N1—C1—C2	123.6 (3)	C14—C15—C18	119.0 (4)
N1—C1—H1	118.2	C16—C15—C18	124.0 (4)
C2—C1—H1	118.2	C17—C16—C15	119.0 (4)
C1—C2—C3	119.4 (4)	C17—C16—H16	120.5
C1—C2—H2A	120.3	C15—C16—H16	120.5
C3—C2—H2A	120.3	N5—C17—C16	123.9 (4)
C4—C3—C2	117.0 (3)	N5—C17—H17	118.1
C4—C3—C6	119.6 (3)	C16—C17—H17	118.1
C2—C3—C6	123.4 (4)	N6—C18—C15	122.2 (4)
C5—C4—C3	120.4 (3)	N6—C18—H18	118.9
C5—C4—H4	119.8	C15—C18—H18	118.9
C3—C4—H4	119.8		
			
N3—Ag1—O1—N2	8.3 (3)	O1—N2—C6—C3	178.3 (3)
N5—Ag1—O1—N2	178.7 (2)	Ag2^iv^—N2—C6—C3	13.6 (5)
N3—Ag1—O1—Ag2^iii^	177.47 (17)	C4—C3—C6—N2	−143.2 (4)
N5—Ag1—O1—Ag2^iii^	−12.15 (17)	C2—C3—C6—N2	35.2 (6)
N2^ii^—Ag2—N1—C1	153.9 (4)	C11—N3—C7—C8	1.2 (8)
O1^i^—Ag2—N1—C1	35.0 (3)	Ag1—N3—C7—C8	178.5 (4)
N2^ii^—Ag2—N1—C5	−31.0 (6)	N3—C7—C8—C9	−0.3 (8)
O1^i^—Ag2—N1—C5	−149.9 (3)	C7—C8—C9—C10	−0.3 (7)
Ag2^iii^—O1—N2—C6	−88.6 (3)	C7—C8—C9—C12	177.4 (5)
Ag1—O1—N2—C6	81.8 (3)	C8—C9—C10—C11	0.0 (7)
Ag2^iii^—O1—N2—Ag2^iv^	77.7 (2)	C12—C9—C10—C11	−177.8 (4)
Ag1—O1—N2—Ag2^iv^	−111.96 (18)	C7—N3—C11—C10	−1.6 (7)
N5—Ag1—N3—C11	−73.7 (8)	Ag1—N3—C11—C10	−179.0 (4)
O1—Ag1—N3—C11	55.0 (4)	C9—C10—C11—N3	1.1 (7)
N5—Ag1—N3—C7	109.0 (8)	O2—N4—C12—C9	179.7 (4)
O1—Ag1—N3—C7	−122.3 (4)	C10—C9—C12—N4	−172.9 (4)
N3—Ag1—N5—C17	61.9 (9)	C8—C9—C12—N4	9.5 (7)
O1—Ag1—N5—C17	−66.4 (4)	C17—N5—C13—C14	−0.1 (7)
N3—Ag1—N5—C13	−122.9 (7)	Ag1—N5—C13—C14	−175.6 (4)
O1—Ag1—N5—C13	108.8 (4)	N5—C13—C14—C15	0.2 (8)
C5—N1—C1—C2	−1.4 (6)	C13—C14—C15—C16	−0.4 (7)
Ag2—N1—C1—C2	174.0 (3)	C13—C14—C15—C18	−177.8 (4)
N1—C1—C2—C3	0.4 (7)	C14—C15—C16—C17	0.5 (6)
C1—C2—C3—C4	1.0 (6)	C18—C15—C16—C17	177.8 (4)
C1—C2—C3—C6	−177.4 (4)	C13—N5—C17—C16	0.2 (7)
C2—C3—C4—C5	−1.4 (6)	Ag1—N5—C17—C16	175.6 (4)
C6—C3—C4—C5	177.1 (4)	C15—C16—C17—N5	−0.4 (8)
C1—N1—C5—C4	0.9 (7)	O3—N6—C18—C15	−177.3 (3)
Ag2—N1—C5—C4	−174.2 (3)	C14—C15—C18—N6	178.2 (4)
C3—C4—C5—N1	0.4 (7)	C16—C15—C18—N6	1.0 (7)

Symmetry codes: (i) *x*, −*y*+1/2, *z*−1/2; (ii) −*x*+1, *y*−1/2, −*z*+1/2; (iii) *x*, −*y*+1/2, *z*+1/2; (iv) −*x*+1, *y*+1/2, −*z*+1/2.

### Hydrogen-bond geometry (Å, º)

**Table:**

*D*—H···*A*	*D*—H	H···*A*	*D*···*A*	*D*—H···*A*
O2—H2···O4	0.84	1.81	2.642 (9)	169
O2—H2···O4′	0.84	2.21	2.997 (14)	156
O3—H3···O1^v^	0.84	1.69	2.527 (4)	174

Symmetry code: (v) *x*, −*y*+3/2, *z*+1/2.

## References

[bb1] Barbour, L. J. (2001). *J. Supramol. Chem.* **1**, 189–191.

[bb2] Gao, S. & Ng, S. W. (2012). *Acta Cryst.* E**68**, m1542. 10.1107/S1600536812046107 PMC3588783 23468748

[bb3] Higashi, T. (1995). *ABSCOR* Rigaku Corporation, Tokyo, Japan.

[bb4] Rigaku (1998). *RAPID-AUTO* Rigaku Corporation, Tokyo, Japan.

[bb5] Rigaku/MSC (2002). *CrystalClear* Rigaku/MSC Inc., The Woodlands, Texas, USA.

[bb6] Sheldrick, G. M. (2008). *Acta Cryst.* A**64**, 112–122. 10.1107/S0108767307043930 18156677

[bb7] Westrip, S. P. (2010). *J. Appl. Cryst.* **43**, 920–925.

